# COVID-19 Dynamics: A Heterogeneous Model

**DOI:** 10.3389/fpubh.2020.558368

**Published:** 2021-01-13

**Authors:** Andrey Gerasimov, Georgy Lebedev, Mikhail Lebedev, Irina Semenycheva

**Affiliations:** ^1^Department of Information and Internet Technology, I.M. Sechenov First Moscow State Medical University, Moscow, Russia; ^2^Federal Research Institute for Health Organization and Informatics, Moscow, Russia; ^3^Center for Bioelectric Interfaces, Institute of Cognitive Neuroscience, National Research University Higher School of Economics, Moscow, Russia

**Keywords:** COVID 19, dynamical model, epidemic, quarantine, antiepidemic measures, population immunity

## Abstract

The mathematical model reported here describes the dynamics of the ongoing coronavirus disease 2019 (COVID-19) epidemic, which is different in many aspects from the previous severe acute respiratory syndrome (SARS) epidemic. We developed this model when the COVID-19 epidemic was at its early phase. We reasoned that, with our model, the effects of different measures could be assessed for infection control. Unlike the homogeneous models, our model accounts for human population heterogeneity, where subpopulations (e.g., age groups) have different infection risks. The heterogeneous model estimates several characteristics of the epidemic more accurately compared to the homogeneous models. According to our analysis, the total number of infections and their peak number are lower compared to the assessment with the homogeneous models. Furthermore, the early-stage infection increase is little changed when population heterogeneity is considered, whereas the late-stage infection decrease slows. The model predicts that the anti-epidemic measures, like the ones undertaken in China and the rest of the world, decrease the basic reproductive number but do not result in the development of a sufficient collective immunity, which poses a risk of a second wave. More recent developments confirmed our conclusion that the epidemic has a high likelihood to restart after the quarantine measures are lifted.

## Introduction

We mathematically modeled the COVID-19 epidemic, as opposed to conducting a statistical analysis of the available data, because over the past 50 years no infectious disease has emerged that could be the basis for testing our model. Thus, SARS and Middle East respiratory syndrome (MERS) did not cause global epidemics. Acquired immunodeficiency syndrome (AIDS) is a disease which lasts for a long time and from which there is no recovery. Influenza epidemics also cannot be the correct basis for an analysis since these are either repeated epidemics in a partially immune population or epidemics where there is some cross-immunity. Therefore, in connection with the novelty of COVID-19, we focused on the development of a mathematical model.

We developed the mathematical model and submitted it for publication when COVID-19 was at its early phase. This disease was first identified in the city of Wuhan. The initial cases of COVID-19 were reported in late November 2019 (1). A month and a half after the first reports, on January 15, there were only 41 cases on record. Then, the number of cases grew rapidly (2–5). The number of cases increased by more than 1,000 from January 15 to February 15. Starting from January 2020, China took extreme quarantine measures. In mainland China, incidences of the disease started to decline, but both the number of countries with an infected population and the incidence rate kept increasing (6, 7).

COVID-19 is caused by the virus SARS-CoV-2 (8–10). Clinical manifestations of the disease resemble those of SARS (11, 12). The mortality rate is lower than in SARS but the incidence rate and the total death toll are significantly higher (5, 13–15). The current COVID-19 epidemic differs in several aspects from the previous one caused by SARS, which was finally extinguished (16–21). First, COVID-19 has a higher basic reproductive number, R_0_, than SARS (22–24). Second, in contrast to SARS, causative pathogen transmission in COVID-19 starts before the end of the incubation stage of the disease (25, 26). Third, unlike SARS, many cases of COVID-19 are asymptomatic, but they are accompanied by a spread of causative pathogens (27–29). These features of COVID-19 lower optimism over the belief that the current epidemic could be successfully controlled.

At the time of writing, several issues remain unclear regarding the spread of this pathogen around the globe, the ways to avoid mass morbidity, estimation of the total incidence rate, and the risk that the incidence rate could start growing after the emergency anti-epidemic measures are partially canceled. As noted above, the large number of unknowns regarding the disease motivated mathematical modeling of the disease progression.

To analyze COVID-19 dynamics, we developed a model that accounted for the heterogeneous composition of the human population (30–33), with subgroups affected differently by the disease. Our model explained the data that were available when the model was developed and predicted the epidemic progression in the case that the anti-epidemic restrictions were lifted. The subsequent developments matched the predictions of our model.

## Methods

The proposed dynamical model accounts for the heterogeneity of infection risk across different age groups. This feature of the model is important because the risk of developing COVID-19 strongly depends on patient age (34–36) and because measures against the disease spread include isolation of elderly individuals. Given these factors, it is important that infection risk, α, for different groups is incorporated in the dynamical model.

In our model, *I(*α*,t)* and *S(*α*,t)* are the proportions of infected and susceptible people, respectively, α is infection risk, *t* is time, and *dF(*α*)* is statistical distribution of infection risk across the population. (∫*dF*(α) = 1). For an infinite isolated population, epidemic dynamics is defined by the set of differential equations (37):

(1) $$\begin{array}{l} \begin{array}{c} \frac{dI\left(\alpha ,t\right)}{dt}=\alpha S\left(\alpha ,t\right)\int I\left(\alpha ,t\right)dF\left(\alpha \right)-\beta I\left(\alpha ,t\right)\\ \frac{dS\left(\alpha ,t\right)}{dt}=-\alpha S\left(\alpha ,t\right)\int I\left(\alpha ,t\right)dF\left(\alpha \right)+\gamma \left(1-S\left(\alpha ,t\right)\right) \end{array} \end{array}$$

where *1/*β is average disease duration from the time of infection till the end of pathogen transmission, and *1/*γ is average lifespan for the people with lifelong immunity or average duration of sustained immunity for the people with transient immunity. The relationship between infection risk, α, and the basic reproductive number, *R*_0_, is given by the equation:

(2) $$\begin{array}{l} R_{0}=\frac{\int \alpha dF\left(\alpha \right)}{\beta } \end{array}$$

For an epidemic that continues for several months, we can neglect the term γ(1−*S*(α, *t*)) that defines population renewal. In this case, the dynamical equations can be rewritten as:

(3) $$\begin{array}{l} \begin{array}{c} \frac{dI\left(\alpha ,t\right)}{dt}=\alpha S\left(\alpha ,t\right)\int I\left(\alpha ,t\right)dF\left(\alpha \right)-\beta I\left(\alpha ,t\right)\\ \frac{dS\left(\alpha ,t\right)}{dt}=-\alpha S\left(\alpha ,t\right)\int I\left(\alpha ,t\right)dF\left(\alpha \right) \end{array} \end{array}$$

The disease progression is usually described using discrete daily samples, where the variations of people's activities throughout the day are averaged out. Accordingly, if *J(*α*,k)* is the portion of infected people on day *k* then Equation (4) can be rewritten to have discrete steps:

(4) $$\begin{array}{l} \begin{array}{c} J\left(\alpha ,k+1\right)=\alpha S\left(\alpha ,k\right)\underset{\alpha }{\int }\overset{N-1}{\underset{n=0}{\sum }}J\left(\alpha ,k-n\right)dF\left(\alpha \right)\\ S\left(\alpha ,k+1\right)=S\left(\alpha ,k\right)-\alpha S\left(\alpha ,k\right)\underset{\alpha }{\int }\overset{N-1}{\underset{n=0}{\sum }}J\left(\alpha ,k-n\right)dF\left(\alpha \right) \end{array} \end{array}$$

where *N* is disease duration in days from the infection onset till the cessation of pathogen transmission, and *R*_0_ = *N*∫α*dF*(α).

Note that *J* cannot be greater than 1. Indeed, *J*(α, *k* + 1) ≥ 0 if *J* ≥ 0, *S* ≥ 0 for any value of *k* or α. This follows from the first Equation in (5) because the right part of the equation is an integral of the numbers that are greater or equal to zero. Then, the sum of both Equations in (5) yields *S*(α, *k* + 1) + *J*(α, *k* + 1) = *S*(α, *k*) ≤ *S*(α, *k*) + *J*(α, *k*) ≤ 1. The fact that S≥0 follows from the equation:

(5) $$\begin{array}{l} S\left(\alpha ,k+1\right)=S\left(\alpha ,k\right)-\alpha S\left(\alpha ,k\right)\underset{\alpha }{\int }\overset{N-1}{\underset{n=K}{\sum }}J\left(\alpha ,k-n\right)\\ dF\left(\alpha \right)\leq S\left(\alpha ,k\right)-\alpha S\left(\alpha ,k\right)\left(1-S\left(\alpha ,k\right)\right). \end{array}$$

Indeed, in our case, α ≤ 1, so *S* does not exceed zero. For the cases where α is >1, the sampling rate could be increased.

The Equations 1, 3, and 4 belong to the susceptible–infected–recovered (SIR) class of models of an epidemic process (38). As followers from the model name, population members can be in one of three states: susceptible, infected, and immune. The susceptible-exposed-infectious-recovered (SEIR) models describe the initial period of an epidemic more accurately (39, 40). In these models, an additional state is added, called exposed, that corresponds to the very start of an infection. This state corresponds to the sterile period when, after being infected, a person does not infect others. The following equation describe the SEIR dynamics:

(6) $$\begin{array}{l} \begin{array}{c} J\left(\alpha ,k+1\right)=\alpha S\left(\alpha ,k\right)\underset{\alpha }{\int }\overset{N-1}{\underset{n=K}{\sum }}J\left(\alpha ,k-n\right)dF\left(\alpha \right)\\ S\left(\alpha ,k+1\right)=S\left(\alpha ,k\right)-\alpha S\left(\alpha ,k\right)\underset{\alpha }{\int }\overset{N-1}{\underset{n=K}{\sum }}J\left(\alpha ,k-n\right)dF\left(\alpha \right) \end{array} \end{array}$$

where *K* is the duration of sterile period in days, and *R*_0_ = (*N* − *K*) ∫ α*dF*(α).

Our model of COVID-19 contains several additional assumptions. First, we assume that, during the initial stage of the disease, its incidence increases exponentially in the non-immune population. COVID-19's growth rate is significantly higher than that of SARS. At the initial stage of the SARS epidemic in 2003, the number of cases tripled during the month of April from 2,000 to 6,000 (41, 42). By contrast, in the second half of January 2020, the number of cases in Wuhan tripled in 3–4 days (i.e., a 40% increase per day). The rate of infection growth depends on *R*_0_ and the disease duration—the factors that affect the distribution of time intervals between sequential infections. The lower limit for the time interval between infections is the time from infection onset till the beginning of virus shedding, and the upper limit is the sum of the interval from infection onset till the end of shedding and the duration of pathogen preservation in the external environment.

Quantifying the time interval between infections is difficult even for well-studied infectious diseases. This is because the beginning of the causative pathogen shedding does not always coincide with the end of the incubation period. Additionally, the time interval between infections is affected by factors such as changes in the intensity of the causative pathogen shedding at different stages of the disease, changes in patient behavior, and person-to-person variability. Because of these unknowns, we based our model on a simplified assumption that during the entire infectious period the infection rate remains constant, and the duration of infectious period, *t*_1_, is equal to the duration of sterile period, *t*_2_: *t* = *t*_1_= *t*_2_. We performed modeling for different values of *t*.

## Results

We used our dynamical model to assess two factors that affect the epidemic progression: (1) the anti-epidemic measures designed to decrease the disease spread, and (2) the accumulation of collective immunity, especially in the high-risk groups.

Table 1 shows how the daily growth in the number of infection cases depends on *R*_0_ and *t*. The estimation of *R*_0_ is only an approximate of the daily growth because of the imprecise values of the sterile and infectious periods and because of the changes in time of virus shedding by an infected person and his/her interactions with other people. Additionally, infection control measures result in a decrease in the number of people interacting with the infected person. For example, the daily growth was 25% in Moscow at the beginning of the COVID-19 epidemic, and it decreased to 15% after the introduction of quarantine measures.

**Table 1:** Daily increase in the number of infection cases at the epidemic's initial stage as the function of reproduction number, *R*_0_, and the duration of sterile/infectious period, *t*.

***R_**0**_***	***t*****, days**			
	**3**	**4**	**5**	**6**
2	19.2%	17.1%	15.3%	13.9%
3	32.5%	28.8%	25.9%	23.5%
4	42.9%	38.0%	34.2%	31.1%
5	51.7%	45.8%	41.2%	37.5%
6	59.3%	52.6%	47.3%	43.1%

**Figure 1:**
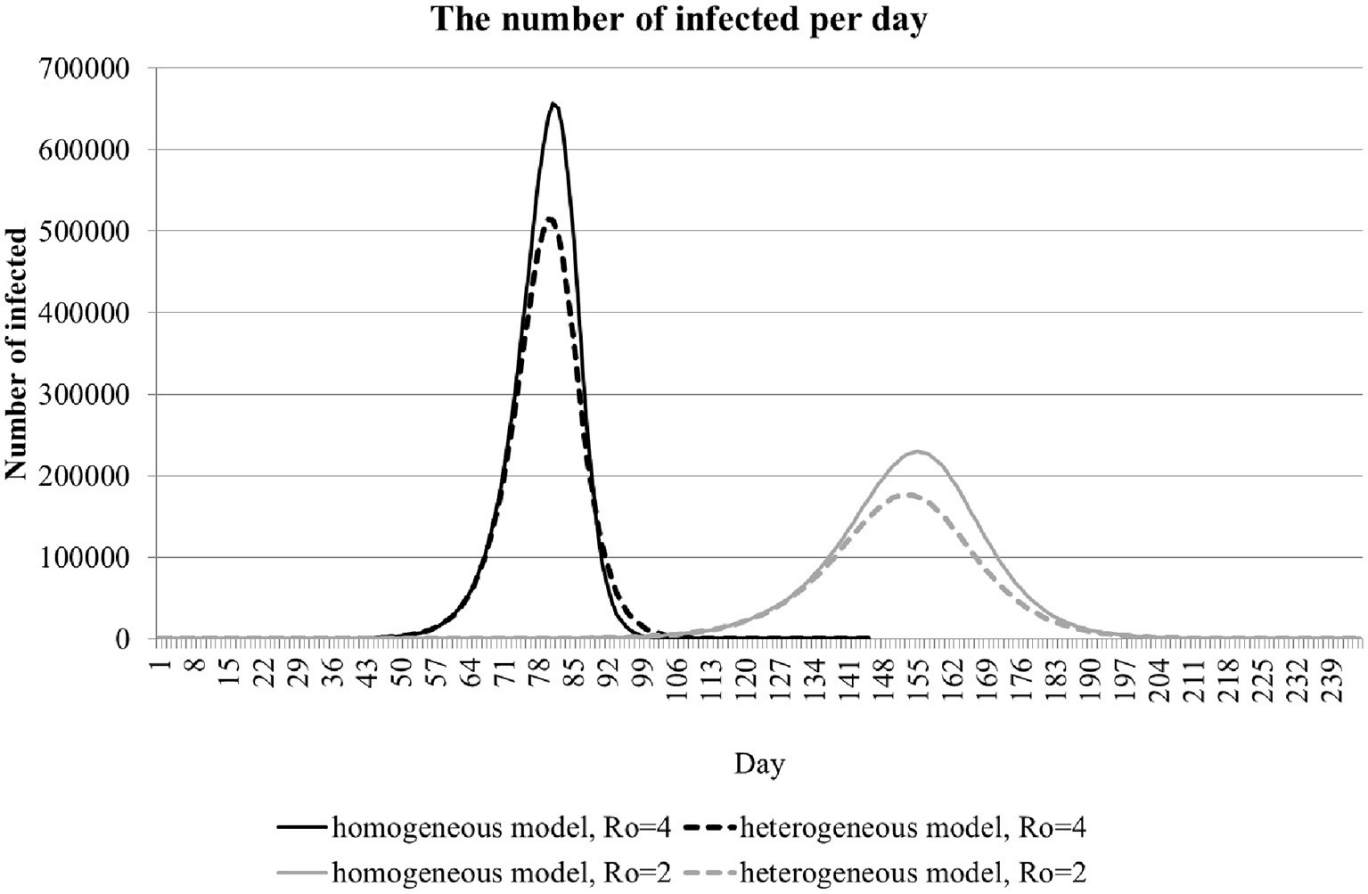
The dynamics of the number of infections per day in a population of 10 million. The curves for homogeneous (solid lines) and heterogeneous (dashed lines) are shown for *R*_0_ equal to 2 and 4.

Figure 1 shows the number of infected people as a function of time for a city with 10 million inhabitants; *t* is set to 5 days, and *R*_0_ is set to 2 or 4. Here the results of a homogeneous model (solid lines) are compared with the results of a heterogeneous model (dashed lines). In the heterogeneous model, infection risk has a uniform distribution between 0 and 2*R*_0_. It is evident from this analysis that the overall incidence rate is lower when the heterogeneity factor is incorporated in the model. A noticeable slowdown in the incidence rate, however, is manifested only when the overall incidence rate has reached a sufficiently high value.

Anti-epidemic measures strive to reduce the COVID-19 infection rate even before it starts to naturally decrease because a substantial portion of the population (including hidden cases) are affected. We modeled the effect of anti-epidemic measures by decreasing *R*_0_ from 4 to 2 (Figure 1). With these settings, anti-epidemic measures of moderate intensity shift the peak in incidence rate forward in time and reduce the peak amplitude. The total incidence does not change appreciably, as evident from the widening of the curve.

**Figure 2:**
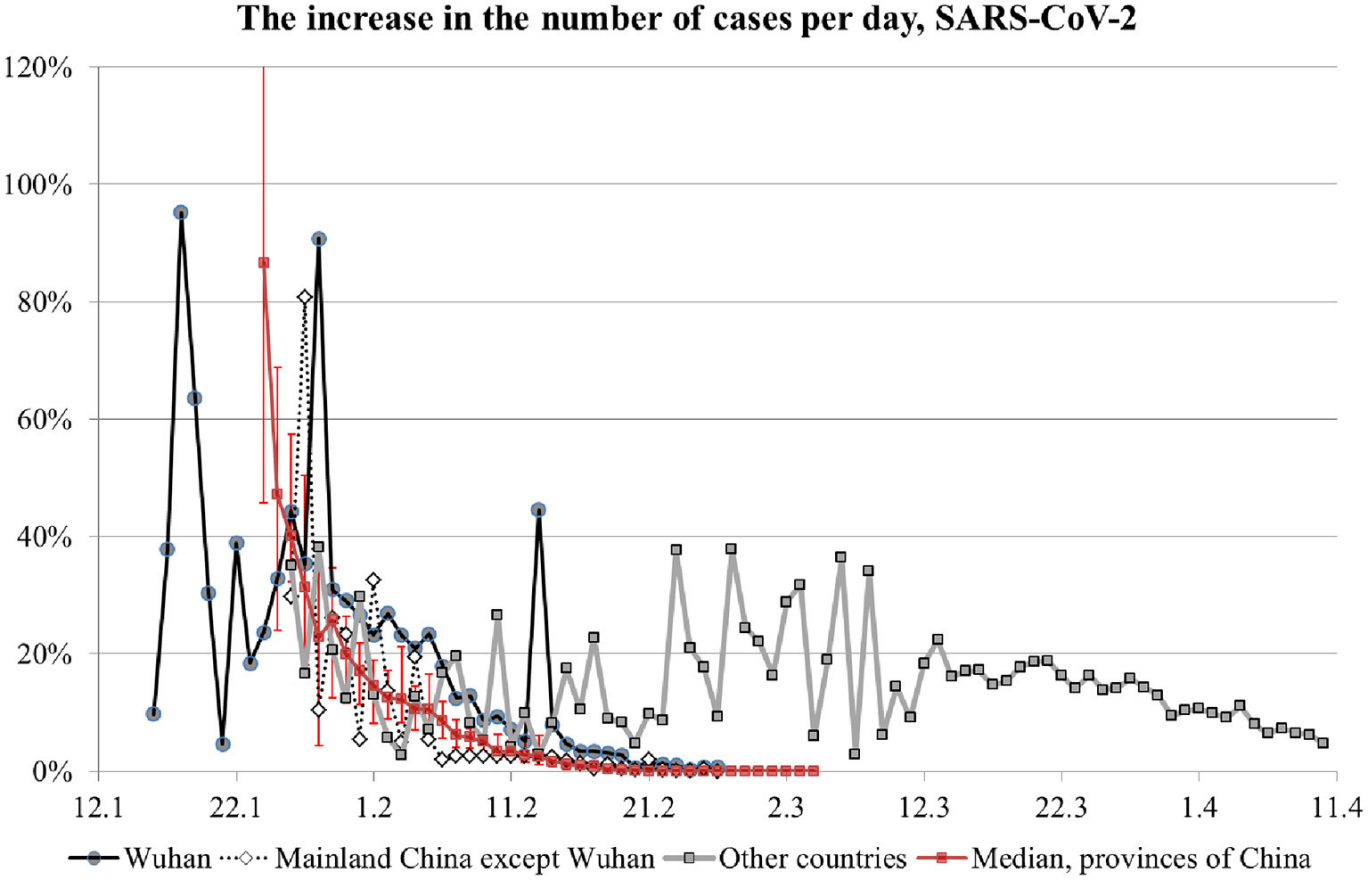
Incidence rate growth rate in different regions.

Ideally, the selection of appropriate anti-epidemic measures should be based on the quantification of *R*_0_ early in the epidemic. One can estimate *R*_0_ based on the disease duration and the growth of incidence rate in the beginning of an epidemic, when the growth is exponential (Table 1). During the exponential-growth stage, daily increase in the total number of cases is constant when expressed as the ratio of the number of cases on a given day to the cumulative sum of cases for the preceding day. Figure 2 shows the dynamics of this ratio for several regions, including China's provinces and other countries. Points are median values, and error bars on the curve for China's provinces (red line) are quartiles. The value of 100% corresponds to the number of infected people doubling on a given day.

For Wuhan's data, the early 90% peak in growth rate is unreliable and can be disregarded because it corresponds to the very beginning of the disease diagnostics with very low samples. For subsequent data with more reliable measurements, the growth rate peaked at 40%, which corresponds to *R*_0_ of 4 (see Table 1), and then decreased to 20% (i.e., *R*_0_ of 2.5) in early February and clearly terminated in mid-February. Given the relatively low overall number of infections, this marked slow-down of infection progression occurred because of the anti-epidemic measures, not because of an accumulation of collective immunity. A note should be made about the 45% surge in growth rate in Wuhan on February 13. It is related to a change in the methodology for calculating the number of cases. On that day, the cases previously considered as questionable were added to the report. Thus, the graph for Wuhan matches the prediction of our model where *R*_0_ and the number of infections decrease because of anti-epidemic measures. A similar dynamic is seen for the rest of the world, where anti-epidemic measures were also undertaken and resulted in the growth rate decrease after March 13.

## Discussion

In this study, conducted during an early stage of the COVID-19 epidemic, we used a heterogeneous model to simulate the epidemic dynamic. With the heterogeneous model, we obtained more accurate results compared to the simpler, homogeneous models. Heterogeneity is an important factor for most infectious diseases. For example, for COVID-19, there is a population of individuals who are infected but do not show noticeable symptoms (20, 25–29). Asymptomatic individuals could be omitted from the medical reports. These people would transmit the infection to others and obtain specific immunity at the end of their infection period. These cases could be underreported because polymerase chain reaction (PCR), the existing methodology for diagnostics, cannot detect the individuals that recovered from the disease. Additionally, there is a bias toward testing mostly the patients with clinical symptoms. Furthermore, there is an age-related heterogeneity as the disease incidence increases with age (35, 43–45). The average age of patients with clinical symptoms is over 50 years old, whereas there are virtually no reported cases of infected children—a distribution that is at odds with the typical risk of infection for airborne infections, which is typically high for all age groups.

Our model accounts for the heterogeneity of infection risk and provides an estimate of the number of infections needed to accumulate for the epidemic to slow down. The model also allows us to assess the effect of anti-epidemic measures. We looked at two factors that reduce the epidemic's growth: (1) anti-epidemic measures, and (2) accumulation of a sufficiently large number of recovery cases from the illness in any form, including the recovery from a mild form without pronounced clinical symptoms. We modeled the first factor by decreasing *R*_0_ and found that the epidemic progression slowed. The total number of eventually infected people, however, remained unchanged. This result brings importance to the second factor, which can guarantee that an infection has ended and would not restart.

The comparison of our model results with the data for Wuhan and the rest of the world indicates an *R*_0_ of 4 at the start of the epidemic, followed by a decrease to 2.5 after the introduction of anti-epidemic measures, and finally a cessation of epidemic growth when the measures become strict. However, the total number of infected people is relatively low at this point, which could be insufficient for the second factor to guarantee that the epidemic has ended. Note that the decrease in incidence growth is almost the same for all Chinese provinces regardless of the huge discrepancies in morbidity levels among the provinces. For example, by March 4 the number of recorded cases in Hubei Province reached 67,466 while the median number of cases in the other 35 provinces amounted to just 245 cases. Such a dynamic is consistent with anti-epidemic measures taking their effect. Indeed, if the decrease in the growth rate was due to an accumulation of unreceptive cases, the decrease rate in the other provinces would have varied greatly. Moreover, a similar dynamic occurred for the rest of the world.

Based on these results, we concluded that:

1. The characteristics of COVID-19 differ markedly from SARS, which makes it hard to contain the disease spread to an affected territory unless the anti-epidemic measures are strict.

2. In the absence of effective anti-epidemic measures, more than 1% of the population could get infected. Should this happen, most cases will occur over a period of several months, which will cause great problems for the treatment of patients.

3. After lifting the emergency quarantine measures, the epidemic could restart because of an insufficient collective immunity level. This course of events should be seriously considered when “reopening” provinces and countries. The same conclusion was also reached by others (46–54).

During the time this manuscript was under review, the COVID-19 epidemic continued to develop, and the predictions of our model were confirmed. A spatial spread of the epidemic was observed in Asia (55, 56), Europe (57, 58), Africa (59, 60), South America (61), and North America (62–65). Moreover, as predicted by the model, lifting anti-epidemic measures resulted in a second wave of the epidemic across the world, which we are currently witnessing (66–69).

## Data Availability Statement

Publicly available datasets were analyzed in this study. This data can be found here: https://github.com/nytimes/covid-19-data.

## Author Contributions

AG, GL, and IS developed the model. AG, GL, ML, and IS interpreted the results and wrote the manuscript. All authors contributed to the article and approved the submitted version.

## Conflict of Interest

The authors declare that the research was conducted in the absence of any commercial or financial relationships that could be construed as a potential conflict of interest.
